# Effects of Digital Cognitive Behavioral Therapy for Insomnia on Self-Reported Sleep Parameters: Systematic Review and Meta-Analysis

**DOI:** 10.3390/clockssleep7040069

**Published:** 2025-12-08

**Authors:** Ingrid Porto Araújo Leite, Viviane Akemi Kakazu, Lucca Andrade Teixeira de Carvalho, Sergio Tufik, Gabriel Natan Pires

**Affiliations:** 1 Departamento de Psicobiologia, Universidade Federal de São Paulo, São Paulo 04024-002, Brazil; ingrid.porto@unifesp.br (I.P.A.L.);; 2 Faculdade Focus, Cascavel 85801-050, Brazil; 3 Instituto do Sono, Associação Fundo de Incentivo à Pesquisa, São Paulo 04020-060, Brazil; 4 Hospital Israelita Albert Einstein, São Paulo 05652-900, Brazil; 5 Faculdade Israelita de Ciências da Saúde Albert Einstein, São Paulo 05653-000, Brazil

**Keywords:** sleep, digital therapeutics, cognitive-behavioral therapy, consumer sleep technology, insomnia, smartphone

## Abstract

Digital Cognitive Behavioral Therapy for Insomnia (dCBT-I) is an effective alternative to therapist-delivered CBT-I. However, there is a lack of meta-analyses assessing its effects on other sleep-related outcomes. We aimed to conduct a meta-analysis of randomized controlled trials (RCTs) evaluating dCBT-I in adults with insomnia through polysomnography (PSG) and sleep diary. Systematic searches were performed in PubMed and Web of Science. The outcomes considered were total sleep time (TST), sleep onset latency (SOL), sleep efficiency (SE), wake after sleep onset (WASO), and number of awakenings (NWAK). Meta-analyses were performed using random-effects models to compare dCBT-I with active (in-person or telehealth CBT-I) or inactive (waiting list, no treatment, or minimal intervention) control groups. Of the fourteen RCTs included, only three employed an active control. As no trials used PSG, the analyses relied solely on sleep diary data. DCBT-I showed no statistically significant differences from active controls, indicating comparable effects with therapist-delivered CBT-I. In contrast, it demonstrated statistically significant effects against inactive controls; TST increased by 0.20 h, SOL decreased by 15.53 min, SE improved by 7.91%, WASO reduced by 15.61 min, and NWAK decreased by 0.53. Future research should prioritize comparisons with therapist-delivered CBT-I and incorporate PSG for measuring these parameters.

## 1. Introduction

Insomnia is a significant public health issue, being among the most prevalent sleep disorders. Approximately one-third of adults worldwide experience insomnia symptoms, with 4 to 22% meeting the diagnostic criteria for insomnia disorder, averaging around 10% [1]. Cognitive Behavioral Therapy for Insomnia (CBT-I) is recognized as the gold-standard treatment according to current guidelines. The main components commonly used in CBT-I protocols, which typically take four to eight 60–90 min sessions, consist of psychoeducation, sleep hygiene, stimulus control, sleep restriction, cognitive therapy, cognitive restructuring, and relaxation techniques [2,3,4,5,6].

CBT-I was originally developed to be delivered by a trained healthcare professional, ideally a board-certified psychologist. However, access to CBT-I remains limited for a significant portion of the population due to the scarcity of trained professionals and the high costs associated with the intervention [7]. Digital CBT-I (dCBT-I) has emerged as a viable and accessible alternative to CBT-I. Although its definition varies, it is widely understood as the adaptation of a therapist-delivered CBT-I program for digital and interactive platforms (most usually smartphone apps), intended for self-guided use by the patient, without the need for a medical setting or the intermediation of a healthcare professional [8].

DCBT-I has already been recognized as a valid and recommended treatment for insomnia by recent guidelines [2,3,5], and its efficacy has been consistently demonstrated in a substantial number of studies [9,10]. However, most of them focused on its effects on symptom severity, usually taking the Insomnia Severity Index (ISI) score as the main outcome. Therefore, there is a lack of meta-analyses about dCBT-I focusing on other sleep-related outcomes, including total sleep time (TST), sleep onset latency (SOL), sleep efficiency (SE), wake after sleep onset (WASO), and number of awakenings (NWAK).

To address this gap, the present article aimed to analyze these sleep parameters through a systematic review and meta-analyses of randomized controlled trials using both sleep diary and polysomnography data.

## 2. Methods

This article is based on an adapted format of the traditional systematic review methodology, referred to as a rapid review, which follows similar steps but employs abbreviated and simplified methods to expedite the review process [11,12]. This design is justified by two main reasons: 1. It is a secondary analysis of data from the latest edition of the Brazilian Guidelines on the Diagnosis and Treatment of Insomnia in Adults (hereinafter “parent study”), whose full methodology has been published elsewhere [2]. The Brazilian guidelines were based on a systematic review of its own, meaning that most of the traditional steps of the systematic review methodology were already taken in the parent study. 2. Several systematic reviews on this subject have already been conducted, strictly following complete protocols and assessing aspects such as risk of bias and publication bias [10,13]. Although our review focused on different outcomes compared with previous meta-analyses, no substantial novelty was expected in procedural aspects common to most RCTs about dCBT-I. Therefore, a rapid review approach with specific methodological adaptations was adopted. It was based on the parent study’s search results, with additional criteria applied to exclude articles not relevant to the aims of this study, without any further risk of bias or publication bias assessments. We then extracted the outcome data of interest, and performed the meta-analyses. The subsections below further detail both the important aspects of the methods employed in the parent study (which are completely detailed elsewhere [2]), and the methods specific to this review. This article was prepared according to the “Preferred Reporting Items for Systematic Reviews and Meta-Analyses” (PRISMA) statement [14].

### 2.1. Search Strategy and Article Selection Process

In the parent study, systematic searches were conducted in the PubMed and Web of Science databases using a search strategy covering two main domains: 1. Insomnia and 2. CBT-I. The retrieved records were imported into the Covidence platform, where duplicates were automatically removed. All records were evaluated in two phases: 1. Titles and abstracts screening, 2. Full texts analysis. In both phases, each record was analyzed by two independent reviewers and conflicts were resolved by a third reviewer.

Additional criteria regarding the specific aims of this study were applied to the resulting dataset, leading to a final selection that comprised only research focusing on dCBT-I and sleep parameters. To be included, each article had to meet the following criteria:

- Abstract and Language: English or Portuguese.
- Article Type: Randomized controlled trials (RCTs).
- Population: Adults diagnosed with insomnia disorder based on the ICSD-3 (International Classification of Sleep Disorders, 3rd edition), DSM-5 (Diagnostic and Statistical Manual of Mental Disorders, 5th edition), or equivalent guidelines. Adults with moderate to severe insomnia symptoms, as measured by the Insomnia Severity Index (ISI) or the Athens Insomnia Scale (AIS), were also included. Self-reported insomnia diagnosis, or symptoms evaluation including mild or subclinical insomnia were excluded.
- Intervention: CBT-I delivered through digital platforms (websites or smartphone applications) for at least 6 weeks.
- Control Group: Active (telehealth CBT-I, in-person CBT-I, or pharmacological treatment) or inactive (waiting list, no treatment, minimal intervention, or placebo) control groups.
- Outcomes: Sleep parameters (calculated from sleep diaries data or obtained through polysomnography).

### 2.2. Data Extraction

The following data were extracted from each article:

- Sleep parameters: TST, SOL, SE, WASO, and NWAK, in any measurement unit (most likely time or percentage).
- Data for each parameter: Extracted for both experimental and control groups, including the number of participants per group, means, and standard deviations. When data was reported in the median, standard error or 95% confidence intervals (95CI), the values were converted into mean and standard deviations following recommended Cochrane formulas.
- Intervention time: The total duration of the intervention until outcome measurement.
- App/site: The name of the application or website used to deliver dCBT-I.
- Comparator: Whether the control group was active or inactive, along with what was specifically used.

### 2.3. Data Synthesis and Analyses

The data analysis in this study was divided into two sections. The first provided a sample description and qualitative synthesis of the selected studies. The second presents a quantitative synthesis, including the meta-analyses, that were performed in two different conditions: dCBT-I vs. active control group and dCBT-I vs. inactive control group.

In this article, the unit of analysis is the comparison (or experiment) between a dCBT-I intervention and a control group. Some articles may have conducted more than one experiment with different participants in the same article. In these cases, the experimental data were collected individually, thus the number of analyses may exceed the number of articles included.

Since outcomes from all studies in each meta-analysis were measured using the same unit of measure, effect sizes were measured as mean difference. Due to the expected variability between the studies, combined effect sizes in the meta-analyses were estimated using the DerSimonian and Laird random effects model. To assess the heterogeneity between the studies, Cochran’s Q test and the I^2^ index were used. The data were presented in forest plots, expressed as effect size and 95CIs. The individual effect sizes are represented by green squares, whose sizes are proportional to the study weight in the meta-analysis. The estimated effect size for meta-analysis is represented by the black diamond. Results were considered statistically significant when the CIs did not include value zero, with *p* < 0.05. All meta-analyses and forest plots were performed using Cochrane RevMan5.

## 3. Results

### 3.1. Sample Description and Qualitative Synthesis

**Figure 1:**
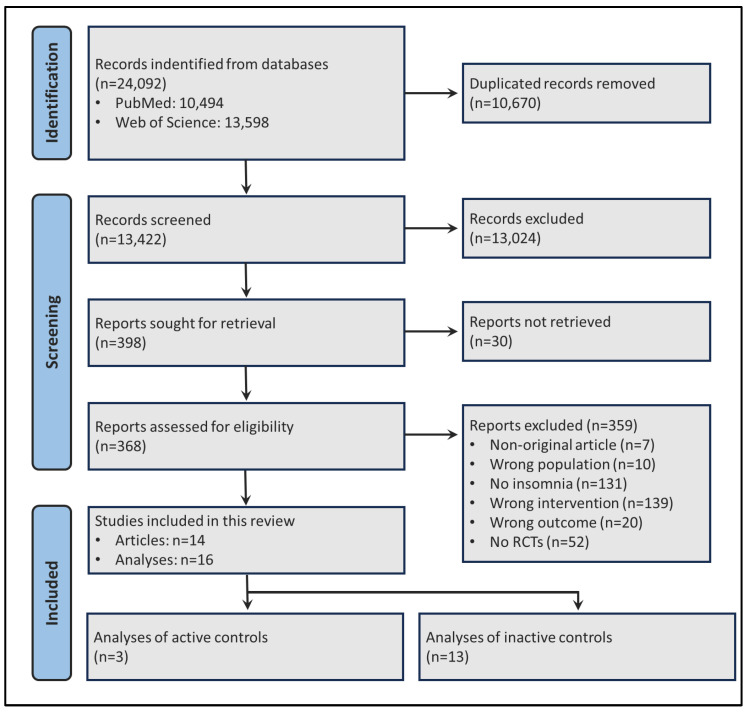
Flowchart of the selection process of articles for the meta-analyses.

A total of 13,422 non-duplicated records were obtained from the initial literature search. After screening and eligibility analyses, 14 articles were included that resulted in a total of 16 analyses (Figure 1 and Table 1). All outcomes were based on self-reported data obtained from sleep diaries, as none of the included records provided PSG data. The most evaluated intervention was SHUTi (Sleep Healthy Using the Internet) (*n* = 5). The most frequently used comparators were no treatment/waiting list (*n* = 6), followed by minimal intervention/sleep education (*n* = 5). The predominant intervention duration was six weeks (*n* = 8).

### 3.2. Quantitative Synthesis

#### 3.2.1. Digital CBT-I Versus Active Control Group

**Figure 2:**
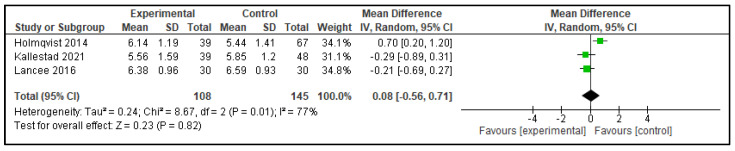
Forest plot of meta-analysis of total sleep time (TST): experimental groups vs. active controls. Articles included: [26,27,28].

**Figure 3:**
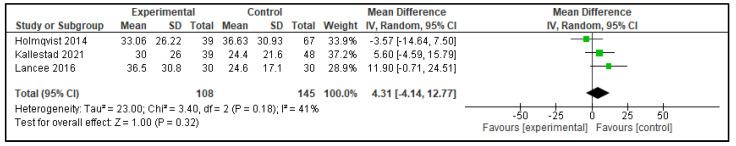
Forest plot of meta-analysis of sleep onset latency (SOL): experimental groups vs. active controls. Articles included: [26,27,28].

**Figure 4:**
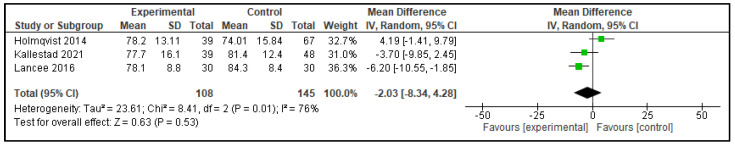
Forest plot of meta-analysis of sleep efficiency (SE): experimental groups vs. active controls. Articles included: [26,27,28].

**Figure 5:**
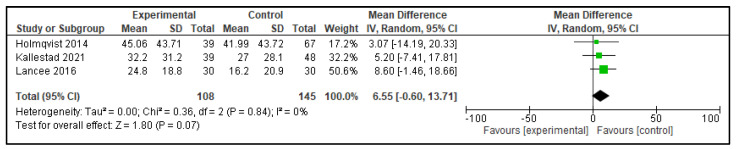
Forest plot of meta-analysis of wake after sleep onset (WASO): experimental groups vs. active controls. Articles included: [26,27,28].

**Figure 6:**
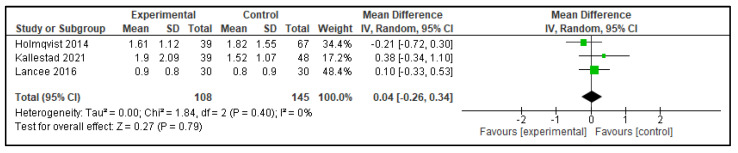
Forest plot of meta-analysis of number of awakenings (NWAK): experimental groups vs. active controls. Articles included: [26,27,28].

Five meta-analyses were performed comparing dCBT-I with therapist-delivered CBT-I (in-person or telehealth), each considering three studies. The following outcomes were analyzed: TST, SOL, SE, WASO, and NWAK. No statistically significant differences were observed in any of the analyses, which indicates that the effects of both interventions on self-reported sleep parameters are equivalent. The forest plots for these analyses are presented in Figure 2, Figure 3, Figure 4, Figure 5 and Figure 6, and the results are summarized in Table 2.

#### 3.2.2. Digital CBT-I Versus Inactive Control Group

**Figure 7:**
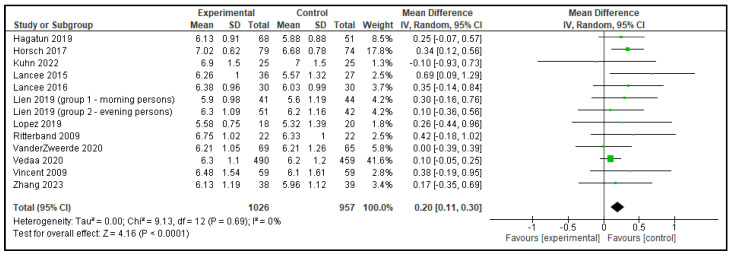
Forest plot of total sleep time (TST): experimental groups vs. inactive controls. Articles included: [15,16,17,18,19,20,21,22,23,24,25,26].

**Figure 8:**
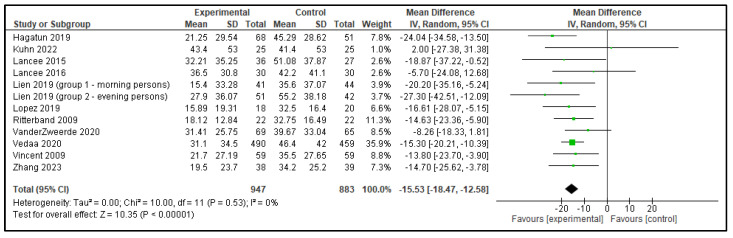
Forest plot of sleep onset latency (SOL): experimental groups vs. inactive controls. Articles included: [15,17,18,19,20,21,22,23,24,25,26].

**Figure 9:**
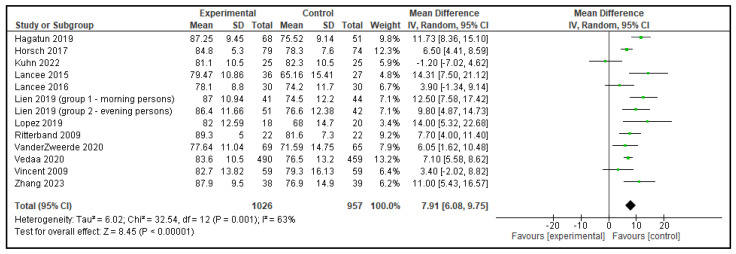
Forest plot of sleep efficiency (SE): experimental groups vs. inactive controls. Articles included: [15,16,17,18,19,20,21,22,23,24,25,26].

**Figure 10:**
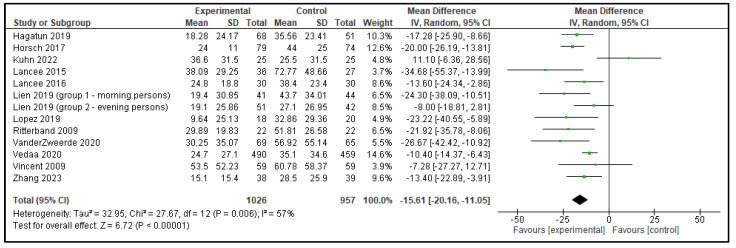
Forest plot of wake after sleep onset (WASO): experimental groups vs. inactive controls. Articles included: [15,16,17,18,19,20,21,22,23,24,25,26].

**Figure 11:**
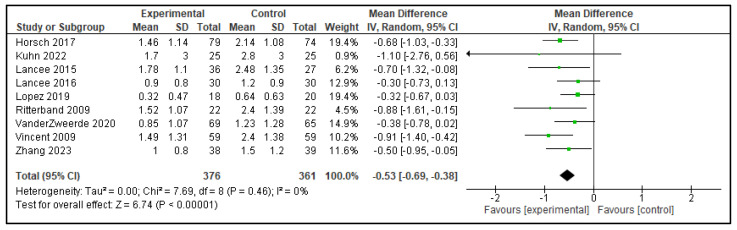
Forest plot of number of awakenings (NWAK): experimental groups vs. inactive controls. Articles included: [16,17,18,20,21,22,23,25,26].

Five meta-analyses were performed comparing dCBT-I with inactive control group, each including 9 to 12 articles. In total, 13 analyses were performed as the study by Lien et al. [15] included 2 distinct groups, which were analyzed separately, as shown in Table 1. The following outcomes were analyzed: TST, SOL, SE, WASO, and NWAK. Statistically significant effects against inactive controls were observed in all outcomes; TST increased by 0.20 h, SOL decreased by 15.53 min, SE improved by 7.91%, WASO reduced by 15.61 min, and NWAK decreased by 0.53. The forest plots for these analyses are presented in Figure 7, Figure 8, Figure 9, Figure 10 and Figure 11, and the results are summarized in Table 2.

## 4. Discussion

In this systematic review and meta-analyses, dCBT-I was found to be effective in improving self-reported sleep parameters. It was equivalent to therapist-delivered CBT-I and showed statistically significant improvement when compared with inactive control group. These results are consistent with the current scientific literature [9,29].

Given the significant impact and high prevalence of insomnia worldwide [1,7,30], assuring the availability of validated and accessible treatments is essential. Online mental health services are becoming increasingly available and have the potential to promote meaningful advancements in healthcare delivery [31]. It is especially relevant as a way to circumvent common limitations of regular psychotherapeutic approaches to insomnia, including high costs and lack of board-certified professionals [32]. DCBT-I is usually regarded as more affordable and more accessible than traditional CBT-I, which grants important practical benefits for digital therapeutics (DTx). As an example of their real-world applicability, internet-based mental health platforms, such as dCBT-I, are integrated into the stepped-care approach in the United Kingdom [33] and are being approved by health regulatory agencies in many countries.

The efficacy of dCBT-I has already been demonstrated in a growing number of studies [34], typically reflected by improvements in insomnia symptoms as the main outcome. However, it is also important to assess its efficacy in other sleep-related outcomes, and our results contribute to this by demonstrating significant effects on self-reported sleep parameters. A deeper understanding of these effects can contribute to a more comprehensive evaluation of this intervention, further reinforcing its clinical relevance and broadening treatment accessibility for a larger portion of the population.

Some methodological aspects of this review increase the reliability of the acquired results. Firstly, only RCTs were included, and observational data were not considered, which increases the certainty of evidence. Secondly, only studies based on clinically relevant cases of insomnia were included (either by using standardized diagnostic criteria or moderate to severe symptoms). Studies encompassing mild insomnia symptoms or self-reported insomnia were not included, which could inflate results possibly due to placebo effects. Thirdly, all studies were derived from an expert guideline, the 2023 Brazilian Guidelines on the Diagnosis and Treatment of Insomnia in Adults [2].

Certain considerations are needed in order to properly contextualize and understand the applicability of the findings of this study. These include aspects related to 1. the external validity to cases of insomnia comorbid with other conditions, 2. the scarcity of RCTs comparing dCBT-I with effective treatments for insomnia, 3. and the lack of PSG as an outcome measurement tool in the included RCTs.

In terms of external validity, it should be noted that the included studies are limited to cases of non-comorbid insomnia (i.e., insomnia not associated with other clinical conditions). Although focusing on non-comorbid insomnia is a relevant approach for generating evidence, it might not reflect the actual clinical profile of many patients with insomnia, who often present with associated clinical conditions (e.g., depression, hypnotic abuse, and pain). The effectiveness of dCBT-I in cases in which insomnia is comorbidly presented with other conditions still warrants further investigation. The psychotherapeutic intervention for these patients is often more complicated and requires a personalized and customized approach, which might be better achieved by means of therapist-delivered CBT-I, rather than by DTx.

Regarding the scarcity of RCTs comparing dCBT-I with effective treatments for insomnia, it is important to highlight that only three among the fourteen included studies employed therapist-delivered CBT-I as the control group, while the others relied on inactive controls (e.g., waiting list or minimal intervention). As there are already several treatments approved for insomnia (both psychotherapeutic and pharmacological) the evaluation of any new treatment should be made not simply in comparison to ineffective interventions. The comparison to waiting list groups or minimal intervention might be adequate to evaluate the efficacy of a given treatment, but only RCTs comparing dCBT-I to other approved treatments would provide relevant information for clinical implementation, such as comparable effectiveness, safety, and cost-effectiveness. Also, considering the nature of insomnia symptoms, the likelihood of placebo effects due to the perception of treatment (especially in studies relying on waiting list control groups and before-after trials) is not negligible. Studies sponsored by dCBT-I developers (which are usually used for validation and registration at regulatory agencies) are most often based on comparison to inactive control groups [35]. This finding appears to be related to “digital exceptionalism”, a term employed in the debate on whether DTx should be regulated with gold-standard evidence and the fact that this is not currently being done. It is important to apply greater rigor in the validation of these treatments to ensure that they are reliable and to protect patients from harms and ineffective treatments [36]. Therefore, it is highly recommended that further RCTs on dCBT-I compare it with therapist-delivered CBT-I, the gold-standard treatment.

Finally, the lack of PSG as an outcome measurement tool in the included RCTs might be considered a limitation, given that it is the gold-standard method for the evaluation of these sleep parameters [37,38]. Our aim in the current systematic review was to evaluate the outcomes based on both subjective and objective data, but no article employing PSG as an outcome measurement was included. Therefore, our results cannot be taken as a definitive demonstration of the effects of dCBT-I on these sleep parameters, as all major RCTs available to date are exclusively based on self-reported outcomes. Nevertheless, they provide clinically relevant information regarding patients’ perceptions of the treatment. A few RCTs have employed PSG in their inclusion criteria, but none have utilized it as an outcome assessment tool. Many RCTs list the lack of PSG as a limitation of their own methodological design [17,39], and possible explanations include 1. the high costs and procedural burden of implementing PSGs for outcome assessment and 2. the fact that the diagnosis of insomnia is clinical and does not require objective sleep assessment.

## 5. Conclusions

Our meta-analyses demonstrated that dCBT-I is effective in improving self-reported sleep parameters, showing significant effects on TST, SOL, SE, WASO, and NWAK. It was found to be comparable to therapist-delivered CBT-I and superior to inactive controls, highlighting its potential as an accessible and effective alternative when conventional therapy is unavailable. To enhance these findings, future RCTs evaluating the efficacy of dCBT-I should prioritize gold-standard CBT-I as the comparator and incorporate PSG as an outcome measure to achieve greater precision in assessing these sleep parameters.

## Figures and Tables

**Table 1:** Descriptions and characterization of the included studies.

Article	Analyses (n)	Program (App or Website)	Comparator	Control Type	Intervention Length
Lien 2019 [15]	2	SHUTi	Minimal intervention/sleep education	Inactive	9 weeks
Horsch 2017 [16]	1	Sleepcare	No treatment/waiting list	Inactive	6 weeks
Van der Zweerde 2020 [17]	1	i-Sleep	Care as usual/waiting list	Inactive	5 weeks
Zhang 2023 [18]	1	Resleep	Minimal intervention/sleep education	Inactive	6 weeks
Hagatun 2019 [19]	1	SHUTi	Minimal intervention/sleep education	Inactive	6 months
Kuhn 2022 [20]	1	Insomnia Coach	No treatment/waiting list	Inactive	6 weeks
Lancee 2015 [21]	1	Digital CBT-I protocol (NFS)	No treatment/waiting list	Inactive	6 weeks
Lopez 2019 [22]	1	Digital CBT-I protocol (NFS)	Minimal intervention/sleep education	Inactive	12 weeks
Ritterband 2009 [23]	1	SHUTi	No treatment/waiting list	Inactive	9 weeks
Vedaa 2020 [24]	1	SHUTi	Minimal intervention/sleep education	Inactive	9 weeks
Vincent 2009 [25]	1	Digital CBT-I protocol (NFS)	No treatment/waiting list	Inactive	6 weeks
Lancee 2016 [26]	2	Digital CBT-I protocol (NFS)	No treatment/waiting list In-person CBT-I	Inactive Active	6 weeks 6 weeks
Holmqvist 2014 [27]	1	Digital CBT-I protocol (NFS)	Telehealth and in-person CBT-I	Active	6 weeks
Kallestad 2021 [28]	1	SHUTi	In-person CBT-I	Active	6–9 weeks

Acronyms: n: Frequency/number of events. NFS: Not further specified. CBT-I: Cognitive Behavioral Therapy for Insomnia. SHUTi: Sleep Healthy Using the Internet (name of the program). Lien 2019 presented two analyses because the participants were separated into two groups: (1) morning people and (2) evening people. Lancee 2016 included two analyses because it had two different types of control groups: (1) inactive control (no treatment/waiting list) and (2) active control (in-person CBT-I). Holmqvist 2014 presented two active control groups for just one experimental group, then the control groups were condensed into one for this analysis.

**Table 2:** Summary of the effects of dCBT-I on self-reported sleep parameters in comparison with either active or inactive control groups.

Outcome	Active Control—Mean Difference (95% CI)	*p*	Inactive Control—Mean Difference (95% CI)	*p*
TST	0.08 (−0.56, 0.71)	*p* = 0.82	0.20 (0.11, 0.30)	*p* < 0.0001
SOL	4.31 (−4.14, 12.77)	*p* = 0.32	−15.53 (−18.47, −12.58)	*p* < 0.00001
SE	−2.03 (−8.34, 4.28)	*p* = 0.53	7.91 (6.08, 9.75)	*p* < 0.00001
WASO	6.55 (−0.60, 13.71)	*p* = 0.07	−15.61 (−20.16, −11.05)	*p* < 0.00001
NWAK	0.04 (−0.26, 0.34)	*p* = 0.79	−0.53 (−0.69, −0.38)	*p* < 0.00001

Acronyms: TST—total sleep time; SOL—sleep onset latency; SE—sleep efficiency; WASO—wake after sleep onset; NWAK—number of awakenings.

## Data Availability

Most data used in this study is publicly available in the previously published Brazilian Guidelines on the Diagnosis and Treatment of Insomnia in Adults. Further data is available upon reasonable request.

## Abbreviations

The following abbreviations are used in this manuscript:

CBT-I	Cognitive Behavioral Therapy for Insomnia
DCBT-I	Digital Cognitive Behavioral Therapy for Insomnia
RCT	Randomized Controlled Trial
PSG	Polysomnography
TST	Total Sleep Time
SOL	Sleep Onset Latency
SE	Sleep Efficiency
WASO	Wake After Sleep Onset
NWAK	Number of Awakenings
NFS	Not Further Specified
